# Bioassay-Guided Fractionation of *Annona macroprophyllata* Seeds to Evaluate Their Anxiolytic and Toxicological Effects

**DOI:** 10.3390/molecules31142517

**Published:** 2026-07-19

**Authors:** Ulises Murrieta-Dionicio, David Martínez-Vargas, María Eva González-Trujano, Gabriel Fernando Moreno-Pérez, Hugo Fernando Narváez-González, Lino Reyes, Holber Zuleta-Prada, Federico del Río-Portilla, Benito Reyes-Trejo

**Affiliations:** 1 Laboratorio de Neurofarmacología de Productos Naturales, Dirección de Investigaciones Biomédicas en Salud Mental, Instituto Nacional de Psiquiatría Ramón de la Fuente Muñiz, Calz. México-Xochimilco 101, Col. San Lorenzo Huipulco, Tlalpan, Ciudad de México 14370, Mexico; murrieta.dionicio.091293@gmail.com (U.M.-D.);; 2 Laboratorio de Productos Naturales, Área de Química, Departamento de Preparatoria Agrícola, Universidad Autónoma Chapingo, Km 38.5 Carretera México-Texcoco, Chapingo 56230, Mexico; hzuletap@chapingo.mx; 3 Laboratorio de Neurofisiología del Control y la Regulación, Dirección de Investigaciones Biomédicas en Salud Mental, Instituto Nacional de Psiquiatría Ramón de la Fuente Muñiz, Calz. México-Xochimilco 101, Col. San Lorenzo Huipulco, Tlalpan, Ciudad de México 14370, Mexico; 4 Hospital de Especialidades Dr. Belisario Domínguez, Av. Tláhuac 4866, San Lorenzo Tezonco, Iztapalapa, Ciudad de México 09930, Mexico; drnarvaezg@hotmail.com; 5 Facultad de Química, Departamento de Química Orgánica, Universidad Nacional Autónoma México, Circuito Escolar S/N, Ciudad Universitaria, Coyoacán, Ciudad de México 04510, Mexico; linoj23@quimica.unam.mx; 6 Instituto de Química, Universidad Nacional Autónoma México, Circuito Escolar S/N, Ciudad Universitaria, Coyoacán, Ciudad de México 04510, Mexico; federico.delrio@gmail.com

**Keywords:** *Annona diversifolia*, anxiety, cyclopeptides, electrocorticogram, plant toxicity, seeds

## Abstract

The genus Annona is scarcely investigated for its neuropharmacological and toxicological activities, especially regarding its seeds. To explore the effects on the central nervous system (CNS) and the acute toxicity (LD_50_) of the polar extracts and fractions obtained from the *A. macroprophyllata* seeds, their potential as a possible source of anxiolytic drugs was evaluated. After determining LD_50_, one or two doses were selected to evaluate the neuronal activity using electrocorticographic (ECoG) recordings, and the anxiolytic-like behavior in mice treated with the crude extracts or some of their fractions was assessed in the experimental models of anxiety, such as the open-field, hole-board, and plus-maze tests. Phytochemical analysis was carried out to identify the most abundant constituents, whose possible mechanism of action was also evaluated by in silico analysis. Results showed CNS depressant activity associated with an anxiolytic-like behavior, in which cherimolacyclopeptide D and squamins C and D were identified. According to a docking analysis, the inhibitory receptors GABA_A_ and 5-HT_1A_ of serotonin are possible mechanisms of action involved in the anxiolytic-like effects of these cyclopeptides, which, in conclusion, might be potential molecules for anxiety therapy.

## 1. Introduction

*Annona macroprophyllata* Donn. Sm. (syn. *Annona diversifolia* Saff.) is a species belonging to the Annonaceae family. It is a tree known in México by local names such as “ilama”, “ilama zapote”, “ilamazapotl” (in Nahuatl), “izlama”, “hilama” or “zapote de vieja” in the states of Colima, Guerrero, and the State of Mexico, while in the Isthmus of Tehuantepec region and the Yucatán Peninsula it is known as “papausa” [1,2]. The fruits of this tree are consumed as food, while its leaves have been used in traditional Mexican medicine for their antidiabetic [3] and anticonvulsant properties [4].

Phytochemical and pharmacological studies have demonstrated that *A. macroprophyllata* can be a source of compounds acting on the central nervous system (CNS). Bio-directed studies using the hexane and/or ethanolic extract of *A. macroprophyllata* leaves led to the isolation of palmitone, an aliphatic ketone with CNS properties. Through behavioral and electrocorticographic (ECoG) analyses, palmitone has been shown to reduce the severity of penicillin-induced seizures in adult rats [4] and to prevent pentylenetetrazol-induced neuronal damage in the CA3 region of the hippocampus in prepubertal rats [5]. Similarly, repeated administration of hexane and palmitone extracts obtained from *A. macroprophyllata* delays the establishment of the kindling state as an experimental model of epilepsy. In addition to its anticonvulsant effects, palmitone exhibits anxiolytic properties. It has been shown to induce anxiolytic-type effects in experimental mouse models with an ECoG profile comparable to those of clinical drugs, acting as a partial or allosteric agonist of the inhibitory 5-HT_1A_ serotonin receptor or the GABA_A_ receptor [6], reinforcing its effect on neurobiological processes.

On the other hand, alkaloids, acetogenins, and cyclopeptides have been identified primarily in the seeds of *A. macroprophyllata*. Some of these have been studied for their antimicrobial [7] and antiprotozoal properties [8,9], and for an interesting antiproliferative cancer activity. In the specific case of *A. macroprophyllata*, the acetogenins identified as Laherradurin and cherimolin-2 exhibited a potential antitumoral inhibitory effect as analyzed in vitro on HeLa and SW-480 cells and in athymic mice bearing these cell lines to demonstrate their antiproliferative cancer activity [10]. Other examples of bioactive compounds actually obtained by bio-guided fractionation are annonacin and muricin P, which reduced intracellular ATP levels and promoted apoptosis and exhibited synergistic in vitro cytotoxicity with sorafenib, a Food and Drug Administration-approved targeted oral therapy used to treat several types of cancer, such as advanced hepatocellular carcinoma [11]. In this context, other cyclopeptides isolated from the *Annona* genus, such as cyclosquamosin B from *A. squamosa*, have shown vasorelaxant activity [12], while cyclosquamosin D [13] and cyclomontanins A, C, and D, obtained from *A. montana* [14], demonstrated inhibitory effects on proinflammatory cytokines in vitro. The anti-inflammatory properties of cyclopeptides have been linked to neuroprotection and improved spatial memory in mice [15], among other activities [16]. Recent research suggests the usefulness of *Annona* seed flour, a novel safe product for human consumption, and demonstrates antimutagenic capacity as a suitable chemopreventive agent, as well as antioxidant and hypoglycemic properties, to be included in functional foods or pharmaceutical formulations [17,18]. However, the potential pharmacological or toxicological effects of natural products from *Annona* seeds on the CNS, particularly those incorporating behavioral and ECoG assessments, have been, or still not, scarcely investigated [19].

In this regard, bio-directed fractionation means a scientific approach in which biological processes, such as plant extracts and biological tests, guide the discovery of chemical compounds with potential biological activity [20]. Thus, it represents a key strategy for identifying fractions responsible for pharmacological and toxicological activities and establishing relationships between chemical composition and biological effects [21]. On the other hand, cyclopeptides possess a closed-loop, ring-shaped structure formed by the union of terminal amino acids. This gives them structural rigidity, making them highly stable, metabolically resistant, with reduced enzymatic degradation, and able to penetrate cell membranes. In this way, cyclopeptides can circulate in the bloodstream, survive the intestinal environment, and cross tissues without being degraded by the protein-digestion, making them interesting compounds for research [8,9,22]. Thus, the anxiolytic-like effects of polar extracts and fractions of *A. macroprophyllata* seeds, using bio-guided fractionation, experimental models of anxiety-like behavior, and ECoG analysis to characterize their effect on neuronal activity in mice, were investigated. Acute toxicity was also calculated to identify the possible therapeutic doses and molecular docking to predict and support the viability of ligand–receptor interaction. As a bioinformatics tool, the computer simulation allows testing chemical compounds of interest against the 3D target structure of the potential inhibitory receptors, such as 5-HT_1A_ of serotonin or GABA_A_ receptors in the present case, to predict whether there is a possibility of effective binding, facilitating rapid digital pre-selection of these compounds for future testing in a biological system [23].

Based on all this information, the hypothesis is that the pharmacological and toxicological effects produced by *A. macroprophyllata* at the central level will depend on the doses and polarity of the components, predicting anxiolytic-like behavior in those fractions enriched with cyclopeptides and a possible action on inhibitory receptors, such as 5-HT_1A_ and GABA_A_, involved in the anxiolytic effects of clinical drugs such as some azapirones and benzodiazepines, respectively.

## 2. Results

### 2.1. Acute Toxicity

The lethal dose fifty (LD_50_) obtained after intraperitoneal (i.p.) administration of the *A. macroprophyllata* extracts or fractions was as follows, in decreasing order of toxicity based on the nature of the extract and number of dead mice:

♦ Ethyl acetate fraction (F-EtOAc) = 10 mg/kg (n = 3 of 6);
♦ Crude methanol extract (MeOH-Ext) = 10 mg/kg (n = 3 of 6);
♦ Methanolic fraction (F-MeOH) = 31.62 mg/kg (geometric mean of the doses causing death from 0% (10 mg/kg, i.p.) and 100% (100 mg/kg, i.p.);
♦ Crude hydroethanolic extract (EtOH-H_2_O-Ext) > 100 mg/kg (n = 0 of 6);
♦ Crude hexane extract (Hex-Ext) = 31.62 mg/kg (geometric mean of the doses causing death from 0% (10 mg/kg, i.p.) and 100% (100 mg/kg, i.p.));
♦ n-Butanol partition (P-BuOH/MeOH) > 100 mg/kg (n = 0 of 6);
♦ n-Butanol partition (P-BuOH/EtOH-H_2_O) > 100 mg/kg (n = 0 of 6).

Mice surviving did not modify their body weight in comparison to the vehicle group through the 14-day evaluation, and no macroscopic tissue alterations were observed after euthanasia. Pharmacological tests were evaluated 30 min after treatment administration; the death of the mice receiving the crude MeOH-Ext (n = 3 of 6 mice) was observed after 24 h and between the 1st and 4th days after administration, while in the case of the F-EtOAc, stated as the most toxic fraction, death occurred after 2 h, so the ECoG recording was evaluated in a period of 90 min. It is worth noting that none of the treatments induced severe ECoG supression or isoelectric activity (EEG flattening) during the initial minutes of treatment or during behavioral anxiety tests—signs typically associated with neurotoxic states or coma, which have previously been observed with other CNS-depressant plant species and with sedative-hypnotic or anesthetic drugs [24].

In a preliminary phase, the doses that produced significant changes in the ECoG were observed; the most toxic fraction (F-EtOAc) was discarded, while the less toxic ones were subjected to fractionation. This allowed us to identify the safest bioactive fractions that maintained an anxiolytic-like behavioral profile in mice during the first 30 min after treatment administration.

### 2.2. Pharmacological Evaluation

#### 2.2.1. Anxiolytic-like Effects

**Figure 1:**
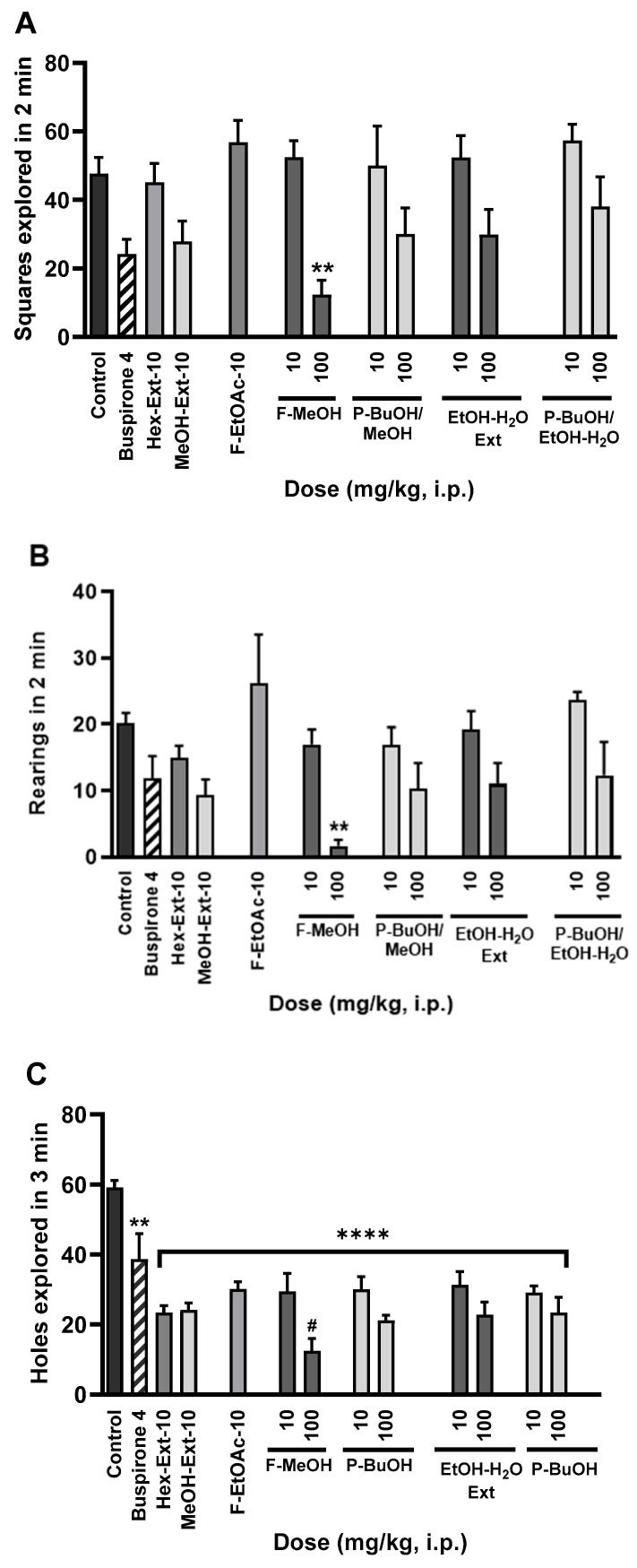
Pharmacological evaluation of the (**A**) ambulatory and (**B**) rearing activities of mice in the open-field test, and (**C**) the number of holes explored in the hole-board test, after i.p. administration of the *A. macroprophyllata* crude extracts or fractions obtained from the seeds compared to the control group. Bars represent the mean ± standard error of 6 repetitions. One-way ANOVA followed by Dunnett’s test; statistical significance at ** *p* < 0.01 and **** *p* < 0.0001. Student’s *t*-test, # *p* < 0.05. Hexane extract: Hex-Ext; methanol extract: MeOH-Ext; ethyl acetate fraction: F-EtOAc; methanolic fraction: F-MeOH; n-butanol partition from MeOH or EtOH-H_2_O extracts, correspondingly: P-BuOH; hydroalcoholic extract: EtOH-H_2_O-Ext.

The polar fraction (F-MeOH), administered at 100 mg/kg, i.p., was the only treatment that affected the ambulatory activity of mice in the open-field test. A significant reduction in both the explored squares (F_12,65_ = 4.59, *p* < 0.0001) and the rearing behavior (F_12,65_ = 3.76, *p* = 0.0002) (Figure 1A and 1B, respectively) was observed.

In marked contrast, all the treatments significantly decreased (F_12,65_ = 9.07, *p* < 0.0001), and in a more pronounced manner than the reference drug buspirone (4 mg/kg, i.p.), the exploratory behavior of mice in the hole-board test. Again, F-MeOH produced more emphatic activity and did so in a dose-dependent manner (t = 2.74, df = 8.73, *p* = 0.024) (Figure 1C).

**Figure 2:**
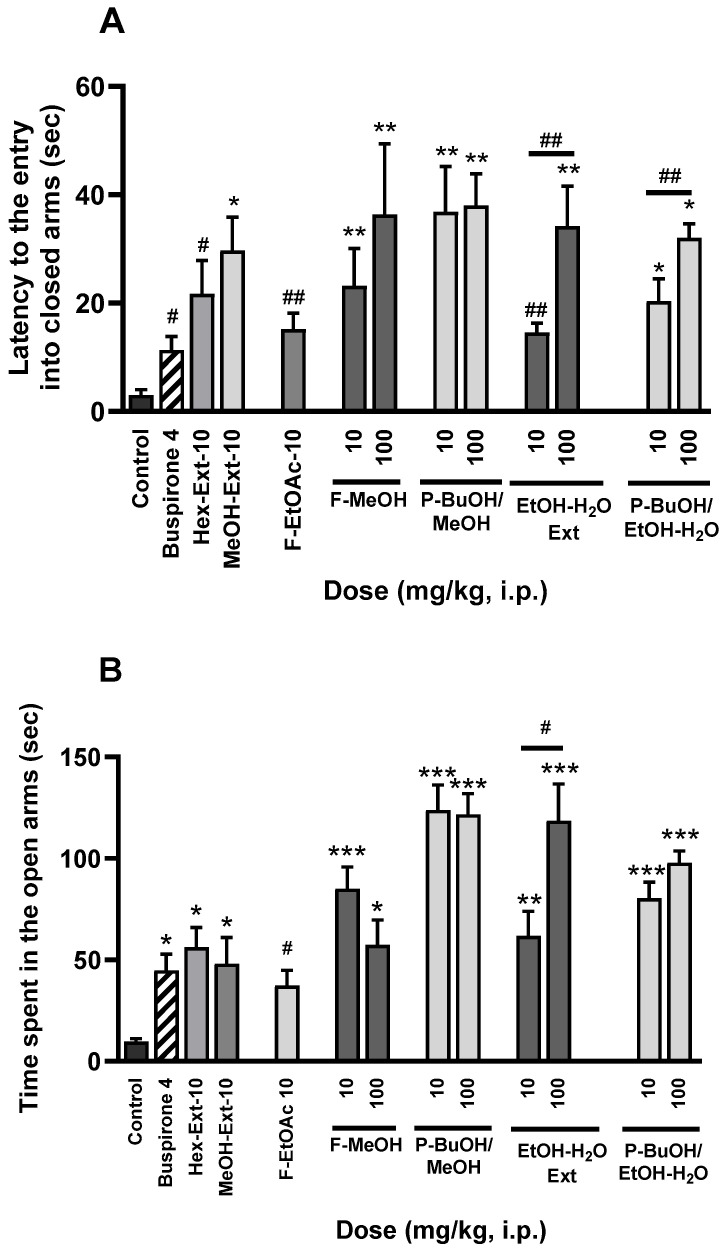
Pharmacological evaluation of the (**A**) latency to entry into closed arms and (**B**) time spent in the open arms in the plus-maze test after i.p. administration of the *A. macroprophyllata* crude extracts or fractions obtained from the seeds compared to the control group. Bars represent the mean ± standard error of 6 repetitions. One-way ANOVA followed by Dunnett’s test; statistical significance at * *p* < 0.05, ** *p* < 0.01, and *** *p* < 0.001. Student’s *t*-test, # *p* < 0.05 and ## *p* < 0.01. Hexane extract: Hex-Ext; methanol extract: MeOH-Ext; ethyl acetate fraction: F-EtOAc; methanolic fraction: F-MeOH; n-butanol partition from MeOH or EtOH-H_2_O extracts, correspondingly: P-BuOH; hydroalcoholic extract: EtOH-H_2_O-Ext.

In the evaluation of the anxiolytic-like activity, the latency to the entry of mice into the closed arms and the time spent in the open arms were assayed in the plus-maze test (Figure 2). In the first case, the fractions produced a major delay in the time to entry into the closed arms, mainly at higher dosage (Figure 2A). In fact, it was observed that in the case of the EtOH-H_2_O-Ext and its P-BuOH at 10 and 100 mg/kg, i.p. showed a response in a dose-dependent manner (t = 2.38 or t = 2.58, df = 8.46 or df = 5.57, *p* = 0.04, respectively) (Figure 2A). Whereas, in the time spent in open arms, all treatments produced a significant response, but a more emphatic result was observed in the case of the fractions (F_12,65_ = 11.05, *p* < 0.0001) as compared to the crude extracts (F_4,25_ = 4.10, *p* < 0.0109), where the F-EtOAc was the less active (t = 3.63, df = 5.43, *p* = 0.013) and the hydroalcoholic extract also showed a dose-dependent effect (t = 2.592, df = 8.672, *p* = 0.03) (Figure 2B).

#### 2.2.2. EcoG Analysis of the Extracts, Fractions, or Partitions of *A. macroprophyllata*

**Figure 3:**
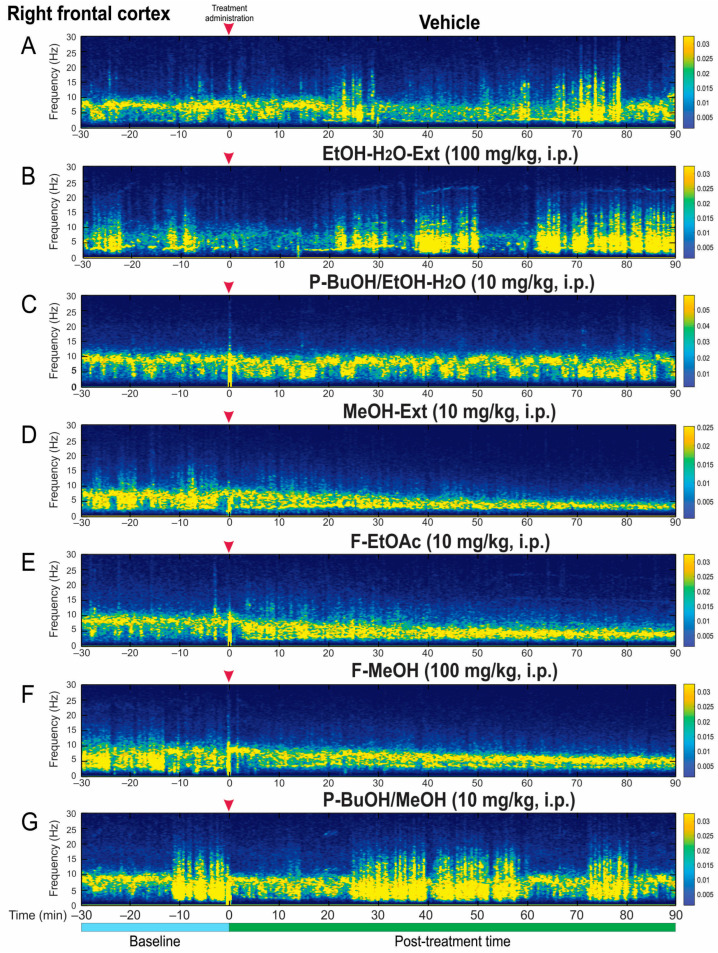
(**A**–**G**) Representative time–frequency spectrograms of ECoG activity from the right frontal cortex. Following treatment exposure, a shift toward increased low-frequency activity was observed after administration of the extracts, fractions, or partitions of *A. macroprophyllata*. Time zero indicates the time point of treatment administration and is marked by red arrowheads. (**A**) Vehicle; (**B**) Hydroalcoholic extract: EtOH-H_2_O-Ext and (**C**) its n-butanol partition (P-BuOH/EtOH-H_2_O); (**D**) methanol extract: MeOH-Ext; (**E**) ethyl acetate fraction: F-EtOAc; (**F**) methanol fraction: F-MeOH; (**G**) n-butanol partition from MeOH extract: P-BuOH/MeOH.

The spectral power analysis of the right frontal cortex revealed clear treatment-related changes from treatment administration up to 90 min post-treatment. Time–frequency spectrograms showed that the extracts, fractions, and partitions of *A. macroprophyllata* induced a marked shift toward low-frequency activity, accompanied by a reduction in higher-frequency activity across the post-treatment period (Figure 3). Mice treated with vehicle exhibited modest changes relative to the baseline period, with spectral power remaining largely distributed at higher frequencies within the theta range, consistent with an active wake state (Figure 3A).

In contrast, administration of EtOH-H_2_O-Ext (100 mg/kg, i.p.) induced a clear redistribution of spectral power toward lower frequency bands, characterized by an increased predominance of shortly delta and beta bands after treatment administration that persisted throughout the post-treatment period (Figure 3B). The P-BuOH/EtOH-H_2_O partition (10 mg/kg, i.p.) primarily promoted shifts toward delta and alpha/beta frequency bands, accompanied by mild and transient fluctuations in higher-frequency bands during the post-treatment period (Figure 3C). In the case of MeOH-Ext (10 mg/kg, i.p.), F-EtOAc (10 mg/kg, i.p.), and F-MeOH (100 mg/kg, i.p.), treatment administration was associated with a pronounced reduction in overall spectral power, resulting in a relative predominance of low-frequency bands and a progressive attenuation of higher-frequency band power across the post-treatment period (Figure 3D–3F, respectively). The P-BuOH/MeOH partition (10 mg/kg, i.p.) produced effects similar to those observed with EtOH–H_2_O-Ext, characterized by intermittent dominance of low-frequency activity and increases in spectral power within the 10–30 Hz range following treatment administration (Figure 3G).

**Figure 4:**
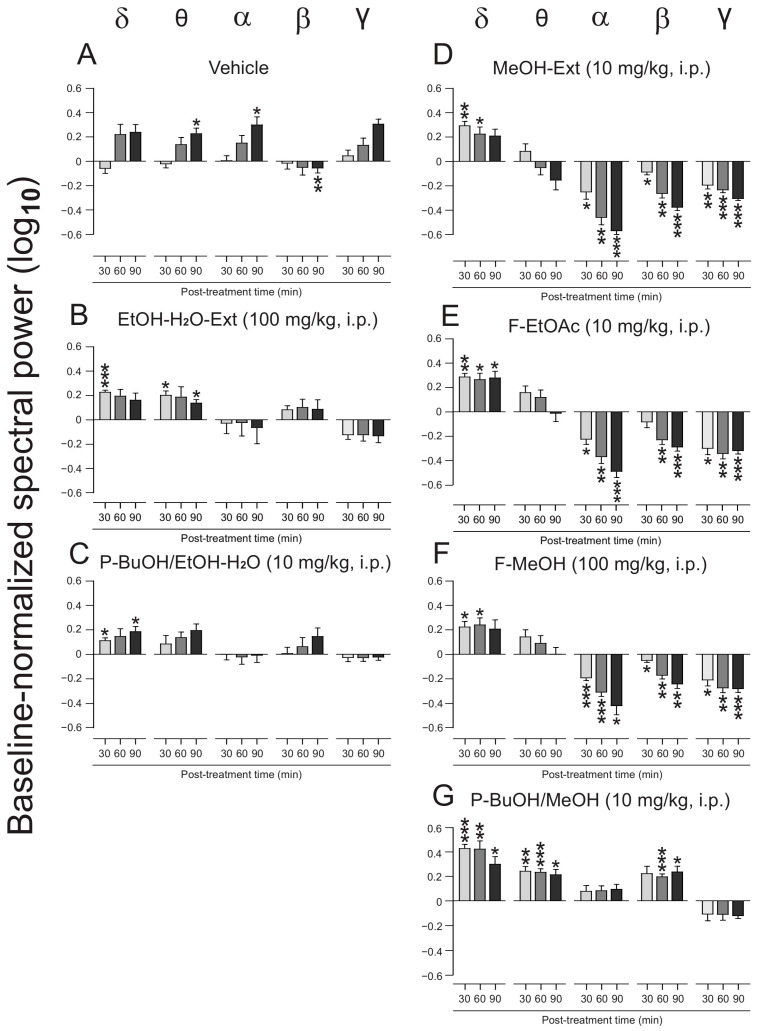
(**A**–**G**) Normalized baseline-related changes in spectral power (log_10_) recorded from the right frontal cortex following the administration of vehicle, extracts, fractions, or partitions of *A. macroprophyllata* in mice. Spectral power changes are shown for the delta (1–4 Hz), theta (4–8 Hz), alpha (8–13 Hz), beta (13–30 Hz), and gamma (30–50 Hz) frequency bands at 30–, 60–, and 90–min post-treatment relative to baseline. Data represent mean ± SEM (6 animals per group). One-way repeated- measures ANOVA followed by Tukey’s test compared with baseline. * *p* < 0.05, ** *p* < 0.01, and *** *p* < 0.001. (**A**) Vehicle; (**B**) Hydroalcoholic extract: EtOH-H_2_O-Ext and (**C**) its n-butanol partition (P-BuOH/EtOH-H_2_O); (**D**) methanol extract: MeOH-Ext; (**E**) ethyl acetate fraction: F-EtOAc; (**F**) methanol fraction: F-MeOH; (**G**) n-butanol partition from MeOH extract: P-BuOH/MeOH.

Consistent with these observations, quantitative spectral power analyses showed significant treatment-dependent effects at 30, 60, and 90 min post-treatment relative to baseline (Figure 4). Vehicle treatment significantly increased theta (F_1.222,6.109_ = 11.32, *p* = 0.013) and alpha (F_1.570,7.851_ = 8.17, *p* = 0.015) band power at 90 min (*p* < 0.05 for both). In addition, beta band power decreased (F_3,15_ = 10.39, *p* = 0.001) at 90 min (*p* < 0.01) (Figure 4A). EtOH-H_2_O-Ext (100 mg/kg, i.p.) induced a significant increase in delta (F_3,15_ = 7.99, *p* = 0.002) and theta (F_3.15_ = 5.27, *p* = 0.01) band power at 30 min (*p* < 0.001 and *p* < 0.05, respectively). Theta band power remained increased at 90 min (*p* < 0.05), whereas alpha, beta, and gamma band power were not significantly affected (Figure 4B). P-BuOH/EtOH-H_2_O (10 mg/kg, i.p.) increased delta band power (F_3,15_ = 4.46, *p* = 0.02) at 30, 60, and 90 min (*p* < 0.05 for all time points) and theta power (F_3,15_ = 4.85, *p* = 0.015) at 60 and 90 min post-treatment (*p* < 0.05 for both) with no significant changes in alpha, beta, or gamma bands (Figure 4C). Similar significant effects were observed following MeOH-Ext (10 mg/kg, i.p.), F-EtOAc (10 mg/kg, i.p.), or F-MeOH (100 mg/kg, i.p.) treatments (Figure 4E,F). Specifically, MeOH-Ext (10 mg/kg, i.p.) increased delta band power (F_3,15_ = 15.75, *p* < 0.001) at 30 and 60 min (*p* < 0.01 and *p* < 0.05, respectively) and decreased alpha (F_3,15_ = 71.92, *p* < 0.001), beta (F_1.978,9.89_ = 78.99, *p* < 0.001), and gamma (F_3,15_ = 57.74, *p* < 0.001) band power at all time points (*p* < 0.05, *p* < 0.01, and *p* < 0.05, respectively) (Figure 4D). F-EtOAc (10 mg/kg, i.p.) increased delta power (F_3,15_ = 23.63, *p* < 0.001) at 30, 60 and 90 min post-treatment (*p* < 0.01, *p* < 0.05, and *p* < 0.05, respectively) and significantly decreased alpha (F_3,15_ = 50.99, *p* < 0.001), beta (F_3,15_ = 35.34, *p* < 0.001), and gamma power (F_1.283,6.413_ = 51.30, *p* < 0.0) at 30, 60, and 90 min (*p* < 0.05, *p* < 0.01, and *p* < 0.001, respectively) (Figure 4E). F-MeOH (100 mg/kg, i.p.) increased delta band power (F_3,15_ = 9.25, *p* = 0.001) at 30 and 60 min (*p* < 0.05 for both times). Conversely, alpha band power was significantly decreased (F_3,15_ = 26.32, *p* < 0.001) at 30, 60 and 90 min (*p* < 0.001, *p* < 0.001, and *p* < 0.05, respectively), while beta (F_1.241,6.203_ = 47.08, *p* < 0.001) and gamma (F_3,15_ = 33.979, *p* < 0.001) band power was decreased across 30, 60 and 90 min (*p* < 0.05, *p* < 0.01, and *p* < 0.01, respectively) (Figure 4F). Finally, P-BuOH/MeOH (10 mg/kg, i.p.) induced significant increases in delta band power (F_3,15_ = 38.41, *p* < 0.001) at 30, 60, and 90 min (*p* < 0.001, *p* < 0.01, and *p* < 0.05, respectively), theta band power (F_1.21,8.53_ = 9.54, *p* = 0.011) (*p* < 0.01, *p* < 0.001, and *p* < 0.05, respectively), and beta band power (F_3,15_ = 16.80, *p* < 0.001) at 60 and 90 min (*p* < 0.001 and *p* < 0.05, respectively) (Figure 4G).

**Figure 5:**
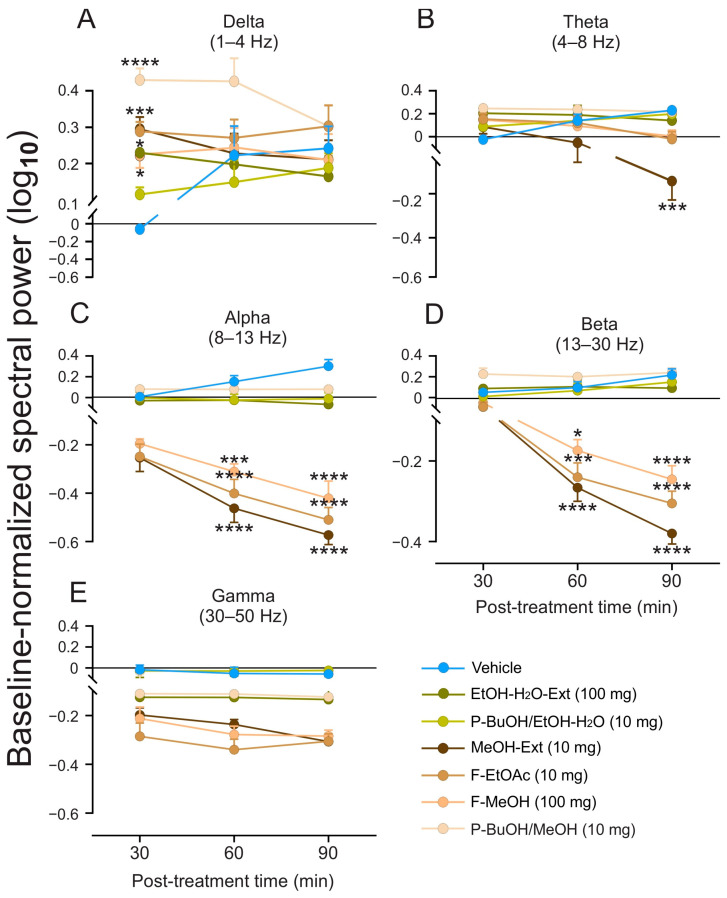
(**A**–**E**) Comparison of treatments on spectral power across frequency bands. Normalized baseline-related changes in spectral power (log_10_) recorded from the right frontal cortex following the administration of vehicle, extracts, fractions, or partitions of *A. macroprophyllata* in mice. Spectral power changes are shown for the (**A**) delta (1–4 Hz), (**B**) theta (4–8 Hz), (**C**) alpha (8–13 Hz), (**D**) beta (13–30 Hz), and (**E**) gamma (30–50 Hz) frequency bands at 30, 60, and 90 min post-treatment relative to baseline. Data represent mean ± SEM of pooled left and right frontal cortices, yielding a total of 8 values per frequency band (6 animals per group). Two-way ANOVA followed by Tukey’s test. * *p* < 0.05, *** *p* < 0.005, and **** *p* < 0.001. Hydroalcoholic extract: EtOH-H_2_O-Ext and its n-butanol partition (P-BuOH/EtOH-H_2_O)*;* methanol extract: MeOH-Ext; methanol fraction: F-MeOH; ethyl acetate fraction: F-EtOAc; n-butanol partition from MeOH extract: P-BuOH/MeOH.

While the previous analyses focused on within-treatment changes over time, a two-way ANOVA was performed to evaluate the effects of treatment, time, and their interaction (Figure 5). For delta band power, a significant treatment × time interaction was observed (F_12,70_ = 4.88, *p* < 0.0001), along with a main effect of treatment (F_6,35_ = 4.16, *p* = 0.0029), whereas no main effect of time was found (F_2,70_ = 1.284, *p* = 0.2833), indicating that temporal changes were dependent on treatment condition. Post hoc comparisons revealed higher spectral power at 30 min following EtOH-H_2_O-Ext (100 mg/kg, i.p.), MeOH-Ext (10 mg/kg, i.p.), F-EtOAc (10 mg/kg, i.p.), and P-BuOH/MeOH (10 mg/kg, i.p.) relative to vehicle (*p* < 0.05 for all comparisons, Figure 5A).

In the theta band, a significant treatment × time interaction (F_12,70_ = 7.10, *p* < 0.0001), as well as main effects of time (F_2,70_ = 3.25, *p* = 0.045) and treatment (F_6,35_ = 3.61, *p* = 0.007) were observed. These effects were driven by a decrease in power at 90 min in the MeOH-Ext (10 mg/kg, i.p.) group compared to vehicle (*p* < 0.001; Figure 5B). A noticeable decrease in spectral power was observed in the alpha and beta bands following treatment with MeOH-Ext (10 mg/kg, i.p.), F-EtOAc (10 mg/kg, i.p.), and F-MeOH (100 mg/kg, i.p.). In the alpha band, significant effects of interaction (F_12,70_ = 6.09, *p* < 0.0001), time (F_2,70_ = 5.71, *p* = 0.005), and treatment (F_6,35_ = 21.70, *p* < 0.0001) were observed. Similarly, in the beta band, significant interaction (F_12,70_ = 5.53, *p* < 0.0001), time (F_2,70_ = 4.02, *p* = 0.0184), and treatment (F_6,35_ = 3.61, *p* < 0.0001) were found. Post hoc comparisons indicated that these decreases occurred at 30 and 60 min compared to vehicle (*p* < 0.05–0.0001; Figure 5C,D). No significant effects were observed for the gamma band across treatments (Figure 5E).

#### 2.2.3. Docking of 5-HT_1A_ Serotonin Receptor and GABA_A_ Receptor Interactions

All three cyclopeptides showed favorable predicted docking scores in both receptor models. In the 5-HT_1A_ receptor, squamin C showed the most favorable predicted affinity in the selected C4 region (ΔG = −10.1 kcal/mol; estimated pKi = 7.40), followed by squamin D (ΔG = −8.7 kcal/mol; pKi = 6.38), and cherimolacyclopeptide D (ΔG = −7.2 kcal/mol; pKi = 5.28) (Table 1).

In the GABA_A_ receptor chimera model, the selected C5 region showed the same qualitative ranking: squamin C (ΔG = −9.9 kcal/mol; pKi = 7.26) > squamin D (ΔG = −9.1 kcal/mol; pKi = 6.67) > cherimolacyclopeptide D (ΔG = −7.3 kcal/mol; pKi = 5.35) (Table 1).

**Figure 6:**
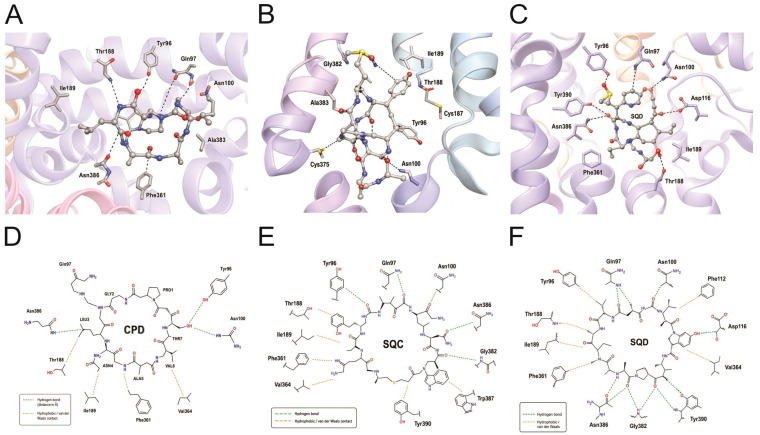
Representative 3D and 2D docking poses of cyclopeptides (**A**,**D**) cherimolacyclopeptide D (CPD), (**B**,**E**) squamin C (SQC), and (**C**,**F**) squamin D (SQD) in the 5-HT_1A_ serotonin receptor, cavity C4, respectively.

**Figure 7:**
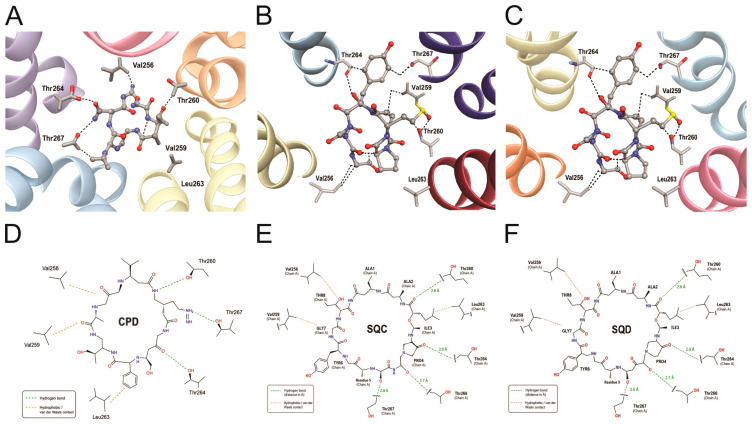
Representative 3D and 2D docking poses of cyclopeptides (**A**,**D**) cherimolacyclopeptide D (CPD), (**B**,**E**) squamin C (SQC) and (**C**,**F**) squamin D (SQD) in the GABA_A_ receptor, cavity C5, respectively.

Figure 6 and Figure 7 show representative 3D (panel A) and 2D (panel B) docking poses for cherimolacyclopeptide D (A, CAPD), squamin C (B, SQC), and squamin D (C, SQD) in the selected cavities of the 5-HT_1A_ serotonin receptor and GABA_A_ receptor. Only the principal interacting residues are labeled to improve readability.

## 3. Discussion

Bio-guided fractionation of *A. macroprophyllata* seeds was performed to identify potential CNS-bioactive compounds. The pharmacological effects and acute toxicity of the extracts, fractions, and partitions were assessed using behavioral tests evaluating anxiety, supported by neuronal activity and predictive docking analysis of the 5-HT_1A_ receptor as a possible mechanism of action. Phytochemical analysis allowed us to characterize neuronal depressant activity associated with anxiolytic-like effects, mainly in the polar extracts, where cyclopeptides such as cherimolacyclopeptide D and squamins C and D were identified. After docking analysis, the involvement of the inhibitory receptors, such as the 5-HT_1A_ of serotonin, was suggested.

First, acute toxicity was determined to decide which doses could be used in the pharmacological screening. It was found that the major acute toxicity was observed after i.p. administration with the crude MeOH extract (LD_50_ = 10 mg/kg, i.p.) compared to the crude non-polar extract (Hex, LD_50_ = 31.62 mg/kg). The crude MeOH extract was separated from a medium polar fraction (F-AcOEt), which had the highest toxicity with an LD_50_ = 10 mg/kg, since mice died in the first 2 h after administration, whereas in the polar fraction (F-MeOH) toxicity decreased, resulting in an increase in the calculated value of the LD_50_ = 31.62 mg/kg. No acute toxicity was obtained with the EtOH-H_2_O-Ext, nor with the partitions obtained with BuOH (P-BuOH/MeOH and P-BuOH/EtOH-H_2_O) at the highest dose tested, suggesting an LD_50_ > 100 mg/kg.

Exposure to various biotic and abiotic factors makes the plant vulnerable to survival. In response, several chemical substances are produced to protect the plant. Such substances, known as secondary metabolites, are of interest in the health area since they are a possible source of new drugs. They can be diverse, and in fact, there is not enough documented information on their pharmacological properties, but less is reported about their toxicological effects [21]. In the case of the Annonaceae family, acetogenins and alkaloids are both chemical groups reported as toxic substances with biological activities found during the early development of the seedling [25]. It is known that the endosperm of mature seeds of *Annona* species contains compounds from different chemical groups, such as essential oils, terpenes, polyphenols, acetogenins, and alkaloids, that act as secondary metabolites for plant defense [26]. Acetogenins are a chemical group present in high amounts and associated with the toxicity of *Annona* seeds of *A. macroprophyllata*. Some examples are laherradurin and rolliniastatin-2, both recognized as the most abundant constituents, which might be because they are biosynthesized simultaneously in the middle stages of the endospermic development [27]. In the case of laherradurin, its acute toxicity in mice was reported as LD_50_ = 11.5 mg/Kg [28]. Whereas liriodenine is an example of an aporphine alkaloid, which is also produced in the early stages of development in the endosperm and seed radicles [29], it has been related to CNS activity by monoamine activity in mice [30] and to neurotoxicity in insects acting on GABA receptors due to the structural similarity to bicuculline, a competitive antagonist of these receptors [31].

In the pharmacological evaluation, corroborated in the ECoG recordings as discussed later, all the extracts and fractions produced depressant activity; only the crude extract MeOH affected ambulatory activity as observed in the open-field test, whereas the most toxic fraction, obtained with EtOAc, showed less activity as observed in the parameter of time spent in the open arms in the plus-maze assay in mice. According to these results, the hydroalcoholic extract and the partitions were the most active treatments that duplicated the anxiolytic-like response with lower toxicity than the crude extracts. These results suggest that anxiolytic-like activity is produced in the presence of different constituents, but polar compounds emphasized this depressant activity as observed after chemical analysis of both P-BuOH partitions obtained from the F-MeOH or hydroalcoholic extract, where the presence of cherimolacyclopeptide D and squamins C-D was found, suggesting their participation in the depressant activity of *A. macroprophyllata* seeds. Early identification of cherimolacyclopeptide D was reported from *A. cherimola* seeds [32] and, recently, from *A. diversifolia* (Syn. *A. macroprophyllata*) by evaluation of its antiprotozoal activity [8,9]. In a similar manner, in the cases of squamin C and D, antiamoeboid [16] and antiprotozoal [8] activities were determined from *A. globifora* and/or *A. diversifolia*, respectively. Thus, this is the first time these cyclopeptides are reported in vivo, in situ, and in silico, providing evidence of their neuronal activity manifested as potential tranquilizing properties.

Spectral power analyses revealed robust, treatment-dependent changes sustained up to 90 min post-administration. Overall, these results indicated a consistent shift toward low-frequency oscillatory activity, accompanied by suppression of higher-frequency bands across several fractions. These results suggest that *A. macroprophyllata* extracts transiently modulate cortical oscillatory dynamics in a frequency-specific manner, with distinct pharmacodynamic profiles across fractions. Thus, according to the in situ ECoG analysis, the extracts and fractions slowed down neuronal activity with significant synchronization of the electrical response, suggesting tranquilizing effects that clearly match the anxiolytic-like behavior observed in the experimental hole-board and plus-maze tests in mice without reaching hypnotic or anesthetic activity. These findings are consistent with electrophysiological studies demonstrating that anxiolytic and sedative compounds induce frequency-dependent electroencephalographic changes, characterized by increased low-frequency power and suppression of higher-frequency activity, reflecting enhanced inhibitory tone, and reduced cortical excitability [33,34,35]. The EtOH–H_2_O extract and the P-BuOH/EtOH–H_2_O partition fraction produced a significant yet moderate increase in delta and theta activity without significant changes in higher-frequency bands, suggesting a CNS-depressant effect associated with anxiolytic-like behavior. In contrast, the crude MeOH extract and the F-EtOAc and F-MeOH fractions generated a more pronounced neurophysiological profile, characterized not only by increased delta activity but also by a significant, marked, and sustained reduction in power within the alpha, beta, and gamma bands. This combination of slow-wave potentiation and fast-frequency suppression has frequently been associated with reduced cortical excitability [24,36,37]. The ECoG profile observed with the extracts or fractions of *A. macroprophyllata* seeds did not fully match that of classic GABAergic sedative or hypnotic drugs. Benzodiazepines such as diazepam—used to treat anxiety disorders, insomnia, and even epilepsy—induce an increase in beta activity (13–30 Hz), a well-established electrophysiological correlate of GABA_A_ receptor allosteric modulation [33,38]. Although it should be noted that increases in delta and theta activity can also occur under certain conditions (particularly at higher doses), these effects are usually accompanied by an increase in beta activity [33]. Other GABAergic modulators, such as sedative and hypnotic drugs like barbiturates (e.g., pentobarbital), agonists like muscimol, and in general anesthetics (e.g., propofol), are characterized by a predominance of low-frequency oscillations alongside the suppression of higher-frequency activity, reflecting reduced cortical activation [36,37]. The profile of the bioactive fractions derived from the seeds of this plant consistently shows CNS depression that does not involve an increase in beta and gamma activity, suggesting that the mechanisms of action may not rely solely on classic GABAergic potentiation. The pattern observed in this study partially aligns with these characteristics, as the absence of a dominant slow-wave state suggests a more moderate level of CNS depression—a finding generally observed with natural products.

Interestingly, this ECoG profile also shares characteristics with non-benzodiazepine anxiolytic agents. Compounds such as buspirone—a 5-HT_1A_ serotonin receptor partial agonist—typically produce a moderate increase in slow-wave activity without a consistent rise in beta activity, reflecting a pharmacological profile distinct from that of classic GABAergic anxiolytics [39]. Similarly, selective serotonin reuptake inhibitors (SSRIs) like paroxetine and fluoxetine are widely used as first-line treatments for anxiety disorders. However, unlike benzodiazepines, they are associated with a delayed onset of therapeutic action and subtle, time-dependent electroencephalographic (EEG) changes, typically without exhibiting a consistent increase in high-frequency activity or a dominant slow-wave pattern [40,41]. In this context, the effects observed with the bio-guided fractionation suggest a differential profile of CNS depression, partly due to the content of chemical compounds and their polarity. The polar extract fractions exhibited lower toxicity than those of medium polarity, displaying a pattern of intense cortical suppression characterized by increased delta activity and decreased power in the alpha, beta, and gamma bands. The high polarity in the hydroalcoholic extract and the n-BuOH partitions produced moderate depressant effects, affecting low frequencies without the generalized suppression of higher-frequency bands. The electropharmacogram analysis in rats has shown that some plant extracts produce similar oscillatory profiles in dose-dependent changes in cortical activity, reflecting their modulatory effects on large-scale neuronal dynamics [42,43]. Pharmaco-EEG fingerprints can be broadly similar to those of anxiolytic drugs such as diazepam. Bioactive compounds isolated from medicinal plants have reinforced the link between electrophysiological modulation and anxiolytic-like behavioral outcomes [6]. These findings support the interpretation that changes in oscillatory dynamics are closely associated with the behavioral effects observed in paradigms such as the hole-board and elevated plus-maze tests. Taken together, the ECoG results show that *A. macroprophyllata* extracts modulate cortical dynamics in a frequency-specific manner, with distinct pharmacodynamic profiles across extracts, fractions, or partitions. The observed slowing of cortical activity and increased synchronization of electrical responses are consistent with a tranquilizing effect, likely mediated by enhanced inhibitory neurotransmission. Notably, these effects occur without evidence of profound cortical depression typically associated with strongly sedative or hypnotic agents, indicating a selective anxiolytic-like profile highlighting their potential as safer alternatives for the management of anxiety-related disorders. This is a preliminary investigation into the pharmacological and toxicological effects of *A. macroprophyllata* seeds on the CNS, which identified the involvement of cyclopeptide-type constituents as potential bioactive compounds. However, further studies are required to elucidate the specific mechanisms of action of the bioactive constituents present in these fractions, as well as the potential anxiolytic response of each cyclopeptide identified.

The in silico results revealed a consistent interaction of the three cyclopeptides with the 5-HT_1A_ serotonin receptors and GABA_A_ receptors. In the 5-HT_1A_ serotonin receptor, squamin C and squamin D displayed broader interaction profiles than cherimolacyclopeptide D, involving aromatic and hydrophobic residues such as Tyr96, Phe361, Trp387, and Tyr390, together with polar residues including Gln97, Asn100, Asp116, Thr188, and Asn386. This interaction pattern is consistent with a receptor region relevant to ligand recognition in the serotonin receptor structural models [44,45]. In the GABA_A_ receptor, squamin C and squamin D shared a conserved C5 interaction environment dominated by Val256, Val259, Thr260, Leu263, Thr264, and Thr267. This shared residue profile supports a conserved predicted binding mode for the squamin-type cyclopeptides. These data provide a feasible molecular hypothesis linking the cyclopeptides to serotonergic and GABAergic targets associated with anxiolytic-like pharmacology. The interaction of squamin C and D with the 5-HT_1A_ serotonin receptor suggests a molecular action consistent with the anxiolytic effects previously reported for *Annona*-derived compounds [46]. Structural studies of serotonin receptors support the importance of ligand binding interactions and 5-HT_1A_ receptor recognition, which is relevant in this study as they are involved in affective and anxiety-related behaviors [47,48]. However, docking cannot establish whether the compounds act as agonists, antagonists, blockers, or allosteric modulators [49]. The docking-derived ΔG, Ki, and pKi values are scoring-function-based approximations, not experimental affinity constants. Therefore, the results should be interpreted qualitatively and as hypothesis-generating. Functional validation, such as receptor-binding assays, Gi/cAMP signaling assays for the 5-HT_1A_ serotonin receptor, and electrophysiological or chloride-flux assays for GABA_A_ receptors, is required to confirm receptor modulation, where molecular dynamics simulations could provide additional information regarding the temporal stability of the ligand–receptor complex, conformational changes, and the potential impact of ligand binding on the receptor interaction site. The potential bioactivity of cyclopeptides has been scarcely investigated, as they have mainly been evaluated for their cytotoxicity using in vitro assays, such as the case of cherimolacyclopeptide D of *A. cherimolia* [31] and some squamins of *A. globiflora* in cancer cell lines [16,50]. Bioactive constituents of *Annona* species with CNS activity have been isolated from the aerial parts, and most have been identified by their alkaloid nature, such as anonaine, lyriodenine, nornuciferine, and 1,2-dimethoxy-5,6,6a,7-tetrahydro-4H-dibenzoquinoline-3,8,9,10-tetraol. Their effects have been associated with monoaminergic activity in murine models of depression [29], which reinforces the potential of species of this genus for CNS disorders, not only anxiety but also depression.

A limitation of the acute toxicity evaluation was the lack of exploration of pharmacokinetic parameters to determine if there was a metabolic influence on the production of toxic metabolites or the range of concentrations causing toxicity, among others. Some mice did not show toxicity immediately, but rather days after administration, except in the case of the F-EtOAc, which caused lethality within the first two hours and was characterized as the most toxic treatment. Another limitation, in this case for the in vivo pharmacological evaluation, was the low yield of the individual compounds identified in the extract, which was insufficient to investigate their possible mechanism of action in mice. Docking analysis carried out allowed predicting that CNS inhibitory receptors such as GABA_A_, interacting with the main inhibitory amino acid in the brain, and the 5-HT_1A_ receptor of the serotonin neurotransmitter might be involved in the depressant activity of these three cyclopeptides identified in the *A. macroprophyllata* extracts contained in its seeds, with a favorable influence on the monoaminergic activity and GABAergic neurotransmission. However, molecular dynamics simulation and functional assays are required to validate the stability and biological relevance of the predicted interactions.

## 4. Materials and Methods

### 4.1. Plant Material

The fruits of *A. macroprophyllata* Donn. Sm. (Syn. *A. diversifolia* Saff.) were acquired in the local market of Tejupilco, State of Mexico, Mexico (18°53′58″ N and 100°09′01″ W) in October 2021. Some specimens were collected in the locality of Plaza de Gallos, Tejupilco, State of Mexico, Mexico (18°51′05″ N and 100°14′38″ W). The specimens (36,509) were identified by botanists from the “Jorge Espinosa-Salas” herbarium-hortorium of the Universidad Autónoma Chapingo (UACh).

### 4.2. Preparation and Fractionation of the Seed Extracts

**Figure 8:**
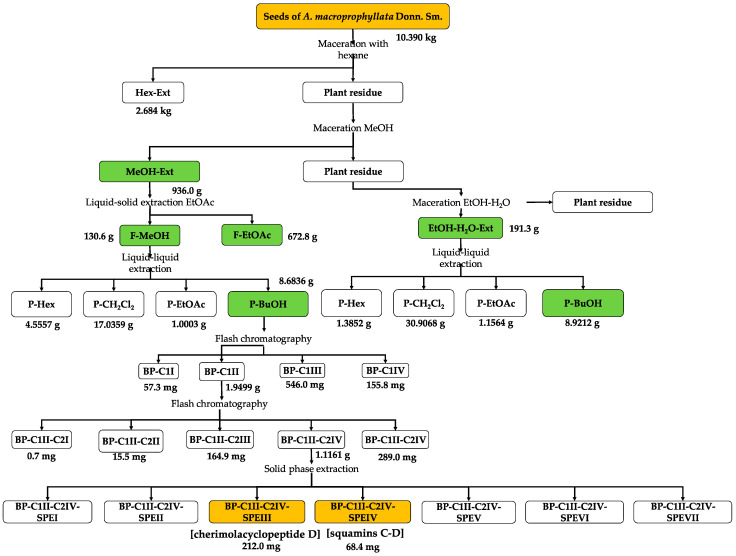
Chemical fractionation of the extracts obtained from *A. macroprophyllata* seeds. Hexane extract: Hex-Ext; methanol extract: MeOH-Ext; methanolic fraction: F-MeOH; ethyl acetate fraction: F-EtOAc; hydroalcoholic extract: EtOH-H_2_O-Ext; hexane partition: P-Hex; dichloromethane partition: P-CH_2_Cl_2_; ethyl acetate partition: P-EtOAc; n-butanol partition: P-BuOH.

The seeds were manually extracted from the fruits, washed with water, and dried in the shade at room temperature for two weeks. After this period, the leathery endocarps were removed using mechanical forceps, and the resulting endosperms were ground in a food processor (NutriBullet, Los Angeles, CA, USA). From 10.3 kg of crushed plant material, successive extraction using the maceration process was carried out with solvents of increasing polarity: hexane (defatted), methanol, and an ethanol-water mixture (1:1, *v*/*v*). Maceration was carried out at room temperature in glass containers, with occasional agitation. For each solvent, a 1:2 (*w*/*v*) ratio and a three-day rest time were used. It was then decanted and filtered to separate the solvent from the plant material. The solvent was recovered under reduced pressure, using a rotary evaporator (R-300 Büchi, Meierseggstrasse, Flawil, Switzerland). Once recovered, it was incorporated back into the plant material up to three extraction cycles for each solvent. Three crude extracts were obtained with the following yields: 2684 g (26.05%) of the Hex-Ext, 936.0 g (9.09%) of the MeOH-Ext, and 191.3 g (1.86%) of an EtOH-H_2_O-Ext (Figure 8).

To eliminate most of the residual lipid fractions of the crude MeOH extract (936.0 g), it was resuspended with EtOAc in a 1:2 (*w*/*v*) ratio. After separation, a soluble fraction and a brown precipitate were obtained. The soluble fraction was concentrated at reduced pressure to obtain 672.8 g of a brownish-green residue (F-EtOAc). On the other hand, the precipitate (263.2 g) was subjected to MeOH washing, and the resulting fraction was evaporated at reduced pressure to obtain 130.6 g of a brown extract (F-MeOH) (Figure 8). The extracts F-MeOH (130.6 g) and EtOH-H_2_O-Ext (191.3 g) were solubilized in 500 mL of distilled water and subjected to a series of liquid–liquid partitions using the following solvents: hexane, dichloromethane, EtOAc, and n-butanol (P-BuOH) at 3 times × 0.5 L, 1:1. After reduced pressure evaporation, the yields of the n-BuOH partitions were 8.68 g from F-MeOH and 8.92 g from crude EtOH-H_2_O extract (EtOH-H_2_O-Ext) (Figure 8).

A sample of 8.09 g of P-BuOH/F-MeOH was dissolved in 15 mL of distilled water and loaded into a SNAP Ultra C18, 30 g cartridge. Fractionation was performed in a Biotage Isolera One (Biotage, Uppsala, Sweden) flash chromatography system with UV absorbance monitoring at 254 and 280 nm. The mobile phase consisted of two solvents: A = H_2_O [0.05% trifluoroacetic acid (TFA)] and B = Acetonitrile (ACN, 0.05% TFA), with a flow rate of 25 mL/min. The cartridge was balanced with 3 column volumes (CV) of 0% B. Elution gradients started with 0% B (5 CV), followed by 0–10% B (3 CV), 10–15% B (4 CV), 15–20% B (3 CV), 20–100% B (3 CV), and 100% B (3 CV). Fractions of 21 mL were collected. The obtained fractions were monitored by thin-layer chromatography (TLC) and revealed with Cl_2_/*o*-tolidine. The fractions that showed similar chromatographic profiles were grouped, and four blocks of fractions designated F-MeOH-BP-C1I-IV were obtained. The F-MeOH-BP-C1II fraction (1.94 g) underwent a second purification by flash chromatography. The sample was dissolved in 5 mL of a CH_2_Cl_2_-MeOH (1:1, *v*/*v*) mixture and loaded into a SNAP Ultra 10 g cartridge. Fractionation was performed in a Biotage Isolera One system (Biotage, Uppsala, Sweden) with UV absorbance monitoring at 254 and 280 nm. The mobile phase was composed of A = CH_2_Cl_2_ and B = MeOH, with a flow rate of 36 mL/min. The cartridge was balanced with 3 column volumes (CV) of 0% B. Elution gradients started with 0% B (1 CV), followed by 0–5% B (2 CV), 5–10% B (3 CV), 10–10% B (5 CV), 10–13% B (6 CV), 13–15% B (5 CV), 15–20% B (5 CV), 20–30% B (4 CV), 30–50% B (2 CV), 50–100% B (2 CV), and 100% B (3 CV). Fractions of 21 mL were collected, monitored by Thin Layer Chromatography (TLC) and revealed with Cl_2_/*o*-tolidine.

**Figure 9:**
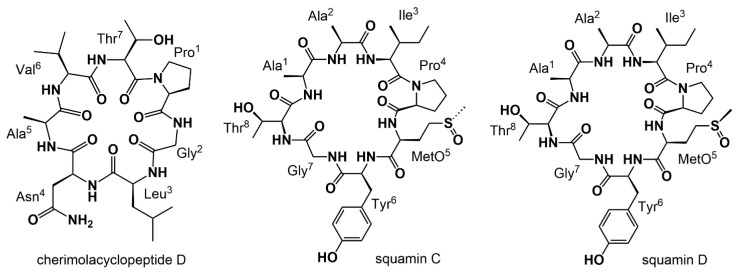
Chemical structures of the cherimolacyclopeptide D, Squamin C, and Squamin D obtained as the most abundant cyclopeptides identified in *A. macroprophyllata* seeds.

The fractions with similar chromatographic profiles were grouped together, obtaining five blocks of fractions, called F-MeOH-BP-C1II-C2I-V. The block of fractions F-MeOH-BP-C1II-C2IV (1.11 g) showed a positive reaction with Cl_2_/*o*-tolidine, so purification was performed by solid phase extraction (SPE). An amount of 1.11 g of F-MeOH-BP-C1II-C2IV fraction was dissolved in 6 mL of H_2_O and loaded into a 10 g HyperSep C18 cartridge (Thermo Scientific, Rockwood, TN, USA) pre-balanced with 50 mL of ACN (0.05% TFA) and subsequently with 50 mL of H_2_O (0.05% TFA). The sample was processed in a vacuum manifold (J.T. Baker, Phillipsburg, NJ, USA), using a mixture of solvents: A = H_2_O (0.05% TFA) and B = ACN (0.05% TFA), with the following gradients: 100% A-0% B (150 mL), 95% A-5% B (200 mL), 90% A-10% B (200 mL), 85% A-15% B (200 mL), 80% A-20% B (200 mL), 70% A-30% B (150 mL), and 100% B (200 mL). Fractions of 10 mL were collected, concentrated at reduced pressure, and monitored by TLC and revealed with Cl_2_/*o*-tolidine. Finally, seven fraction blocks with similar chromatographic profiles were obtained (see Supplementary Materials), called F-MeOH-BP-C1II-C2IV-SPEI-VII. The fractions F-MeOH-BP-C1II-C2IV-SPEIII (212.0 mg) and F-MeOH-BP-C1II-C2IV-SPEIV (68.4 mg) were positive for the reaction with Cl_2_/*o*-tolidine. Both fractions were analyzed by H^1^-NMR, showing a predominant enrichment in three cyclopeptides: F-MeOH-BP-C1II-C2IV-SPEIII (cherimolacyclopeptide D) and F-MeOH-BP-C1II-C2IV-SPEIV (squamin C and squamin D) (Figure 9). These cyclopeptides were identified by comparison of their physical and spectroscopic properties with those previously published, by determining their ^1^H- and ^13^C-Nuclear Magnetic Resonance (NMR, see Supplementary Materials) spectra in one and two dimensions (HSQC, HMBC, COSY, TOCSY, and ROESY) [8].

### 4.3. Nuclear Magnetic Resonance (NMR) Measurements

The NMR experiments were performed using a Bruker AVANCE III HD 700 spectrometer (Bruker BioSpin, Billerica, MA, USA) or an Agilent DD2 400 MHz spectrometer (Agilent Technologies, Santa Clara, CA, USA). All analyses were conducted at 298 K in deuterated solvents. A total of 4 mg of the samples were dissolved in either acetone-*d*_6_ or CD_3_OD. Chemical shifts were reported on the δ scale (in ppm), using tetramethylsilane (TMS, δ = 0.00 ppm) as an internal reference. Coupling constants (J) were expressed in Hertz (Hz) for the ^1^H-NMR spectrum; spectrometer frequency (SF) = 699.95 MHz, and for the ^13^C-NMR spectrum, SF = 176.02 MHz.

### 4.4. Drugs and Reagents

Trifluoroacetic acid (TFA), tetramethylsilane (TMS), acetone-*d*_6_, DMSO-*d*_6_, and *o*-tolidine were purchased from Sigma-Aldrich (St. Louis, MO, USA). Thin-layer chromatography (TLC) was performed using aluminum plates coated with silica gel 60 F254 (Merck, Darmstadt, Germany). Analytical-grade solvents were used in the chromatography and were purchased from Merck (Darmstadt, Germany). The reference drug buspirone was purchased from Sigma-Aldrich (St. Louis, MO, USA). All the treatments were freshly prepared on the day of the experiments and administered intraperitoneally (i.p.) at 0.1 mL/10 g body weight.

### 4.5. Experimental Design

#### 4.5.1. Animals

Swiss-Webster (SW) male mice (25–35 g body weight; 7–8 weeks old) were divided into groups of six animals. The rodents were provided by the vivarium of the Instituto Nacional de Psiquiatría “Ramón de la Fuente Muñiz” (INPRFM), which were placed in acrylic boxes at a controlled temperature (22 °C ± 1 °C) with a 12 h light/dark cycle and fed ad libitum. The experiments were carried out in accordance with the technical guidelines for the production, care, and use of animals in the SAGARPA Mexico laboratory (NOM-062 ZOO-1999) and the approval of the INPRFM research committee (28 September 2007) for projects NC093280.0 and NC093280.1.

#### 4.5.2. Acute Toxicity

Acute toxicity was determined by calculating the lethal dose fifty (LD_50_) in mice receiving a maximal dosage of 100 mg/kg, i.p., of the crude extract or fraction evaluated, followed by doses of 10 or 1 mg/kg, i.p., depending on the dose producing mouse death. According to the Organization for Economic Cooperation and Development (OECD), when there is no information on a substance to be tested, for animal welfare reasons it is recommended to use 300 mg/kg body weight as the starting dose. If test substance-related mortality is produced, further testing at the next lower level may need to be carried out [51]. The maximum initial dose was selected because it was the one that, when evaluated in other parts of the plant, showed significant pharmacological activity [4]. For acute toxicity, attention was directed to the observation of tremors, convulsions, salivation, diarrhea, lethargy, sleep, and coma. At the end of the 14-day observation and registration of the body weight, surviving mice were euthanized for a macroscopic evaluation to examine tissue damage according to the modified report of Lorke. The geometric mean of the doses causing death from 0% and 100% was determined as LD_50_ [52].

#### 4.5.3. Pharmacological Evaluation

##### Anxiolytic-like Effects

Seventy eight mice were divided into 13 groups of n = 6 individuals to receive an intraperitoneal (i.p.) acute dose of one low and/or high dose of the following treatments: (1) Control (vehicle, 0.2–0.5% Tween 80 in distilled water), (2) crude hexane extract (Hex-Ext, 10 mg/kg), (3) crude methanol extract (MeOH-Ext, 10 mg/kg), (4) fraction ethyl acetate (F-EtOAc, 10 mg/kg), (5–6) fraction methanol (F-MeOH, 10 and 100 mg/kg), (7–8) partition with n-butanol (P-BuOH/MeOH, 10 and 100 mg/kg, (9–10) hydroalcoholic extract (EtOH-H_2_O-Ext, 10 and 100 mg/kg), and (11–12) its n-butanol partition (P-BuOH/EtOH-H_2_O, 10 and 100 mg/kg), and finally (13) the reference drug buspirone (BUSP4, 4 mg/kg). Thirty minutes after treatment, mice were exposed to pharmacological evaluation using the following tests:

*Open-field test* [53]. The mice were placed individually in an acrylic box (23 × 40 × 20 cm) whose interior base is divided into 12 squares (9.5 cm × 7 cm). The number of squares explored by each mouse was recorded in a time interval of 2 min. A significant decrease in the number of explored squares, demonstrating a reduction in ambulatory activity, was taken as a reference for a possible sedative effect.

*Hole-board test* [54]. The mice were placed individually on the surface of a wooden box (24 × 24 × 20 cm). This surface is perforated with 16 equally distributed 3 cm diameter holes and is delimited by transparent acrylic walls 20 cm high. The number of times the mouse inserted its head into one of the holes was recorded in a time interval of 3 min. The significant decrease in orifice exploration compared to the control was indicative of an anxiolytic effect.

*The elevated plus-maze test.* To emphasize the anxiolytic-like activity of the treatments, the well-known and widely validated plus-maze test [55] was also included, which consisted of a wooden cross, raised 50 cm from a base, with two open arms (30 × 5 cm) and two closed (30 × 15 × 5 cm), and a central open space (5 × 5 cm) where each mouse was placed. The latency and number of entries into each closed or open arm and the time remaining in each of them were then recorded over a 5-min period.

##### Neuronal Electrical Activity Analysis

To analyze changes in neuronal activity by the effects of the extracts, fractions, or partitions of *A. macroprophyllata* seeds, an in situ ECoG recording was carried out to determine the spectral power in mice as follows:

Stereotaxic surgery, electrocorticographic (ECoG) recording, and spectral power analysis

*Electrode implantation surgery.* Forty-two adult male Swiss-Webster mice (25–35 g, eight weeks old) were anesthetized with isoflurane (4% in O_2_ for induction and 1–2% for maintenance) and placed in a rodent stereotaxic frame (Stoelting, Wood Dale, IL, USA). According to the atlas of Paxinos and Franklin [56], two stainless-steel screws were bilaterally implanted over the frontal cortices (from Bregma: AP + 2.0 mm; ML 1 mm). An additional screw electrode, placed over the cerebellum (AP +5 mm; ML 0), served as a reference. All electrodes were soldered to a connector and secured on the skull with dental cement. After the surgical procedure, animals were allowed one week to recover.

*ECoG recordings.* After recovery, animals were placed in a sound-insulated chamber for 20 min to habituate to the experimental conditions (n = 6 per group). ECoG activity was then recorded for 120 min, a 30-min baseline period followed by 90 min after treatment administration according the following groups: (1) vehicle, (2) MeOH-Ext (10 mg/kg, i.p.), (3) F-EtOAc (10 mg/kg, i.p.), (4) F-MeOH (100 mg/kg, i.p.), (5) P-BuOH/MeOH (10 mg/kg, i.p.), (6) EtOH-H_2_O-Ext (100 mg/kg, i.p.), and (7) P-BuOH/EtOH-H_2_O (10 mg/kg, i.p.). The ECoG signals from both frontal cortices were amplified and bandpass filtered (1–70 Hz) using an electroencephalograph GRASS Model 8-18D (GRASS Instrument Co., Quincy, MA, USA) and digitized at a sampling rate of 500 Hz.

*ECoG spectral power analysis.* The effects of each treatment on ECoG activity recorded from the frontal cortices were analyzed using spectral power analysis. Power spectra were computed using custom-made routines developed in MATLAB R2020a (MATLAB, The MathWorks Inc., Natick, MA, USA), applying the fast Fourier transform (FFT) to ECoG signals in the 1–50 Hz frequency range [6]. FFT was applied to five 60-s ECoG segments obtained during baseline wakefulness, defined by low-amplitude, high-frequency activity and confirmed by visual inspection of the ECoG traces. Additional 60-s segments were analyzed at multiple time points following treatment administration (30–35, 60–65, and 85–90 min). Absolute spectral power was calculated for the delta (1–4 Hz), theta (4–8 Hz), alpha (8–13 Hz), beta (13–30 Hz), and gamma (30–50 Hz) frequency bands for each animal. Power values from each segment were first log_10_-transformed. Baseline log_10_ power was then obtained by averaging the five baseline segments for each animal, channel, and frequency band. Treatment-related changes were quantified by subtracting the mean baseline log_10_ power from the corresponding log_10_-transformed post-treatment values, yielding baseline-normalized changes in spectral power. This normalization approach is widely used in electrophysiological studies to stabilize variance and enable proportional comparisons of spectral power across conditions, thereby facilitating appropriate statistical analyses of drug effects [57,58].

##### In Silico Study

Ligands were prepared as complete cyclic peptide structures and minimized prior to docking using Avogadro v1.2. Receptor files were prepared by removing non-relevant heteroatoms and waters, adding polar hydrogens, and assigning charges. CurPocket-guided cavity screening was used to define candidate receptor cavities (https://cadd.labshare.cn/cb-dock2/; accessed on 1 July 2026). Docking was performed with AutoDock Vina v1.1.2 using the grid centers and box sizes shown in the Supplementary Materials. For each receptor-ligand pair, the selected pose was interpreted based on docking score, biological plausibility, structural comparability across ligands, and interaction consistency. Figures were prepared using Chimera (UCSF ChimeraX v1.18), PyMOL (Molecular Graphics System v3.0, Schrödinger, LLC.), and Discovery Studio Visualizer (BIOVIA Discovery Studio Visualizer: 2025, Dassault Systèmes; accessed on 1 July 2026) [59,60].

Molecular docking was performed to explore a plausible molecular basis for the observed anxiolytic-like effects of the cyclopeptides cherimolacyclopeptide D, squamin C, and squamin D. The 5-HT_1A_ serotonin receptor structure (PDB ID: 7E2Y) and the GABA_A_ receptor chimera model (PDB ID: 5OSA) were retrieved from the Protein Data Bank [61,62,63]. Ligands were modeled as complete cyclopeptides, energy-minimized using the MMFF94 force field in Avogadro [47], converted to PDBQT format, and docked using AutoDock Vina [64]. Receptor preparation included removal of crystallographic water molecules and non-relevant heteroatoms, addition of polar hydrogens, and assignment of Gasteiger charges [65,66].

Potential binding cavities were evaluated using CurPocket-guided screening, followed by docking into the selected cavities. For the 5-HT_1A_ serotonin receptor, cavity C4 was selected for comparative analysis because it provided a biologically plausible and structurally consistent receptor region across cherimolacyclopeptide D, squamin C, and squamin D. For the GABA_A_ receptor, cavity C5 was selected for cross-ligand comparison; although cherimolacyclopeptide D showed a marginally better score in C3, the C5 pose was retained for consistency with squamin C and squamin D. All evaluated cavities, grid centers, and box dimensions are reported in more detail in the Supplementary Materials. Docking-derived Ki and pKi values were estimated from the Vina ΔG scores using ΔG = RT ln (Ki) and pKi = -log10 (Ki) and were used only for qualitative comparison.

#### 4.5.4. Statistical Analysis

Data are expressed as the mean ± standard error of the mean (S.E.M.) of each treatment. To test normal distribution, a Shapiro–Wilk test was applied. Behavioral responses evaluated in the anxiety tests were analyzed by one-way Analysis of Variance (ANOVA) followed by Dunnett’s test, n = 6 repetitions. In the ECoG, baseline-related changes in spectral power were analyzed using one-way repeated-measures ANOVA, with time (baseline, 30, 60, and 90 min post-treatment) as the within-subject factor, followed by Tukey’s post hoc test. To evaluate the effects of treatment, time, and their interaction, a two-way ANOVA was conducted with treatment as the between-subject factor and time as the within-subject factor, followed by Tukey’s post hoc test. When the assumption of sphericity was not met, the Greenhouse–Geisser correction was applied. Statistical analysis was performed using the SPSS statistical software (version 20.0, IBM Corporation, New York, NY, USA) and GraphPad Prism (version 8.0, GraphPad Software Inc., La Jolla, CA). Statistical significance was set at *p* < 0.05.

## 5. Conclusions

It is known that chemical agents used in current disease treatments cause various adverse effects, including some that are toxic, which occur during therapeutic activity. This has driven risk–benefit analysis in patients and pharmacovigilance. Medicinal plants are no exception, and the degree of toxicity sometimes depends on the part of the plant from which the extract is obtained due to the chemical nature of its bioactive compounds. Since extracts prepared from these plants can contain significant amounts of effective and safe bioactive compounds, but also other toxic compounds, it is important to identify them and determine their potential use in human and animal health. As a source of novel compounds for exploring alternative treatments, not only for psychiatric conditions but for all types of diseases, it is important to explore the effects of natural products on the CNS to rule out any neurotoxic damage. Therefore, the objective of this research was to determine acute toxicity, as well as behavioral and physiological changes in brain activity. While toxicity can be determined with a small number of animals, such as 5 or fewer, statistically significant differences in a pharmacological effect might require a larger sample size per dose evaluated, from 6 to 10. In this investigation, an effort was made to maintain the minimum number of animals to comply with OECD guidelines for determining acute toxicity and validating responses in anxiety tests. As a result, and through a bio-guided fractionation strategy, the toxic fractions were separated from those that maintained anxiolytic activity with the greatest safety. The pharmacological and toxicological data included in this report provide evidence of the depressant CNS activity of constituents of *A. macroprophyllata* seeds involving compounds of different chemical nature that produce CNS depressant effect, suggesting the cyclopeptide chemical nature is bioactive, acting on inhibitory receptors like 5-HT_1A_ and GABA_A_, which could be a potential source of therapy for conditions such as anxiety.

## Figures and Tables

**Table 1:** Docking summary for the selected binding cavities.

Receptor	Ligand	Selected Cavity	ΔG (kcal/mol)	Ki	pKi	Key Residues
5-HT_1A_ (7E2Y)	CPD	C4	−7.2	5.28 µM	5.28	Tyr96, Gln97, Asn100, Thr188, Ile189, Phe361, Val364, Asn386
5-HT_1A_ (7E2Y)	SQC	C4	−10.1	39.5 nM	7.40	Tyr96, Gln97, Asn100, Thr188, Ile189, Phe361, Val364, Gly382, Asn386, Trp387, Tyr390
5-HT_1A_ (7E2Y)	SQD	C4	−8.7	420 nM	6.38	Tyr96, Gln97, Asn100, Asp116, Thr188, Ile189, Phe112, Phe361, Val364, Gly382, Asn386, Tyr390
GABA_A_ chimera (5OSA)	CPD	C5 *	−7.3	4.46 µM	5.35	Val256, Val259, Thr260, Leu263, Thr264, Thr267
GABA_A_ chimera (5OSA)	SQC	C5	−9.9	55.4 nM	7.26	Val256, Val259, Thr260, Leu263, Thr264, Thr267
GABA_A_ chimera (5OSA)	SQD	C5	−9.1	214 nM	6.67	Val256, Val259, Thr260, Leu263, Thr264, Thr267

* Cherimolacyclopeptide D (CPD) in the GABA_A_ receptor showed a slightly more favorable score in C3 (−7.5 kcal/mol), but C5 was selected for cross-ligand comparison with squamin C (SQC) and squamin D (SQD). Ki and pKi values are docking-derived estimates and should not be interpreted as experimentally determined binding constants.

## Data Availability

Spectral data and other data that support the findings are available from the corresponding authors upon reasonable request.

## Supplementary Materials

The following supporting information can be downloaded at: https://www.mdpi.com/article/10.3390/molecules31142517/s1, Figure S1: HPLC chromatogram of BP-C1II-C2IV-SPEIII; peak 1 = cherimolacyclopeptide D, peak 2 = unidentified compound, monitored at 230 nm; Figure S2: HPLC chromatogram of BP-C1II-C2IV-SPEIV; peak 1 = unidentified compound, peak 2 = mixture of squamin C + squamin D (co-eluting compounds), monitored at 230 nm; Figure S3: Stacked 1H-NMR spectra of the mixture of squamins C + D (black), compared with pure squamin D (blue) and pure squamin C (green), in acetone-d6 at 700 MHz (pure compound spectra from Murrieta-Dionicio et al., 2024); Table S1: 1H and 13C NMR spectroscopic data for cherimolacyclopeptide D (CD3OD, 298 K); Table S2: Docking cavity screening parameters for all evaluated cavities; Table S3: Principal contact residues used for interpretation and figure labeling; Table S4: Docking-derived Ki and pKi values for selected poses.

## Abbreviations

The following abbreviations are used in this manuscript:

AN	Acetonitrile
LD_50_	Acute toxicity
ANOVA	Analysis of Variance
ATP	Adenosine Triphosphate
BUSP	Buspirone
CPD	Cherimolacyclopeptide D
CNS	Central Nervous System
CV	Column Volumes
EEG	Electroencephalographic
Hex-Ext	Crude Hexane Extract
MeOH-Ext	Crude Methanol Extract
DGIPyS	Dirección General de Investigación, Posgrado y Servicio
F-EtOAc	Ethyl Acetate Fraction
FFT	Fast Fourier Transform
F-MeOH	Methanolic fraction
P-BuOH/MeOH	n-butanol partition from MeOH extract
EtOH-H_2_O-Ext	Crude Hydroethanolic Extract
P-BuOH/EtOH-H_2_O	n-butanol partition from EtOH-H_2_O extract
ECoG	Electrocorticographic
GABA_A_	Gamma amino butyric A
INPRFM	Instituto Nacional de Psiquiatría Ramón de la Fuente Muñiz
i.p.	Intraperitoneal
NMR	Nuclear Magnetic Resonance
PDB	Protein Data Bank
SQC	Squamin C
SQD	Squamin D
TLC	Thin-Layer Chromatography
TFA	Trifluoroacetic acid
TMS	Tetramethylsilane
UAch	Universidad Autónoma Chapingo
