# Supplementary material for: Bioassay-Guided Fractionation of Annona macroprophyllata Seeds to Evaluate Their Anxiolytic and Toxicological Effects
Source: Molecules. 2026 Jul 19;31(14):2517. doi: 10.3390/molecules31142517 (PMC13414508; doi:10.3390/molecules31142517)
Supplement: Supplementary file 1 [file molecules-31-02517-s001.zip › molecules-4418578-supplementary.pdf]

Supplementary material

Bioassay-Guided Fractionation of *Annona macroprophyllata* Seeds to Evaluate Their Anxiolytic and Toxicological Effects

Ulises Murrieta-Dionicio [1,2,†], David Martínez-Vargas [3,†], María Eva González-Trujano [1,*], Gabriel Fernando Moreno-Pérez [1], Hugo Fernando Narváez-González [4], Lino Reyes [5], Holber Zuleta-Prada [2], Federico del Río-Portilla [6] and Benito Reyes-Trejo [2,*]

[1] Laboratorio de Neurofarmacología de Productos Naturales, Dirección de Investigaciones Biomédicas en Salud Mental, Instituto Nacional de Psiquiatría Ramón de la Fuente Muñiz, Calz. México-Xochimilco 101, Col. San Lorenzo Huipulco, Tlalpan, Ciudad de México 14370, Mexico; murrieta.dionicio.091293@gmail.com (U.M.-D.)

[2] Laboratorio de Productos Naturales, Área de Química, Departamento de Preparatoria Agrícola, Universidad Autónoma Chapingo, Km 38.5 Carretera México-Texcoco, Chapingo 56230, Mexico; hzuletap@chapingo.mx

[3] Laboratorio de Neurofisiología del Control y la Regulación, Dirección de Investigaciones Biomédicas en Salud Mental, Instituto Nacional de Psiquiatría Ramón de la Fuente Muñiz, Calz. México-Xochimilco 101, Col. San Lorenzo Huipulco, Tlalpan, Ciudad de México 14370, Mexico

[4] Hospital de Especialidades Dr. Belisario Domínguez, Av. Tláhuac 4866, San Lorenzo Tezonco, Iztapalapa, Ciudad de México 09930, Mexico; drnarvaezg@hotmail.com

[5] Facultad de Química, Departamento de Química Orgánica, Universidad Nacional Autónoma México, Circuito Escolar S/N, Ciudad Universitaria, Coyoacán, Ciudad de México 04510, Mexico; linoj23@quimica.unam.mx

[6] Instituto de Química, Universidad Nacional Autónoma México, Circuito Escolar S/N, Ciudad Universitaria, Coyoacán, Ciudad de México 04510, Mexico; federico.delrio@gmail.com

* Correspondence: evagontru@yahoo.com.mx or evag@inprf.gob.mx (M.E.G.-T.); breyest@chapingo.mx (B.R.-T.)

† These authors contributed equally to this work as first author.

Supplementary material of characterization of cyclopeptides cherimolacyclopeptide D, squamin C, and squamin D.

The cyclopeptide-enriched fractions (BP-C1II-C2IV-SPEIII and BP-C1II-C2IV-SPEIV) were analyzed by high-performance liquid chromatography (HPLC) using a Varian ProStar system. A Phenomenex Jupiter Proteo column (4.6 mm i.d. x 250 mm, 4 µm, 90 Å) was used. Elution was performed in gradient mode using H_2O (0.05 % TFA v/v) (solvent A) − ACN (0.005 % TFA v/v) (solvent B). For the BP-C1II-C2IV-SPEIII fraction, the gradient consisted of increasing solvent B from 5 % to 50 % over 18 min, followed by decreasing it to 5 % over 6 min, at a constant flow rate of 1.0 mL/min. Detection was performed by UV absorbance at 230 nm. For the BP-C1II-C2IV-SPEIV fraction, a gradient of 5 to 40 % B was used over 25 min, followed by a re-equilibration step to 5 % over 5 min, maintaining a flow rate of 1.0 mL/min and monitoring at 230 nm (Figs. S1 and S2, Table S1). The cyclopeptides (cherimolacyclopeptide D, squamin C, and squamin D) were identified by comparing their spectroscopic data (Fig. S3) with those previously reported [8]. Additionally, the presence of cherimolacyclopeptide D was confirmed by structural elucidation based on ^{1}H and ^{13}C Nuclear Magnetic Resonance (NMR) data, as well as two-dimensional experiments (HSQC, COSY, and HMBC), acquired on CD_3OD. Furthermore, the presence of a mixture of squamin C and D was established by comparing the ^{1}H NMR spectra of the sample with previously published data on acetone-d_6 [8].

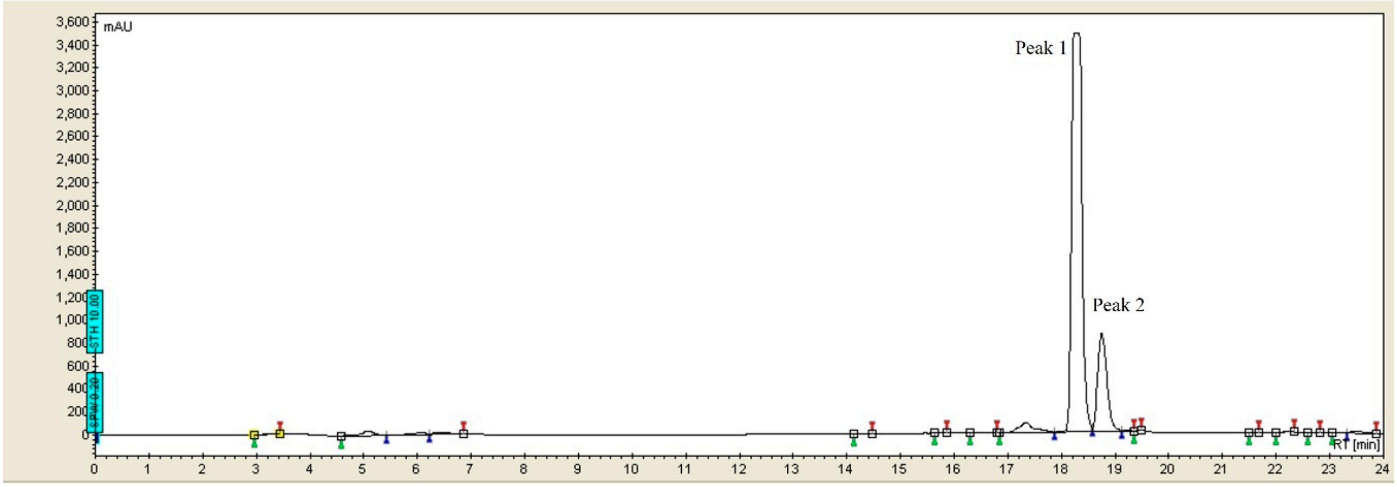

Figure S1. HPLC chromatogram of BP-C1II-C2IV-SPEIII; peak 1 = cherimolacyclopeptide D, peak 2 = unidentified compound, monitored at 230 nm.

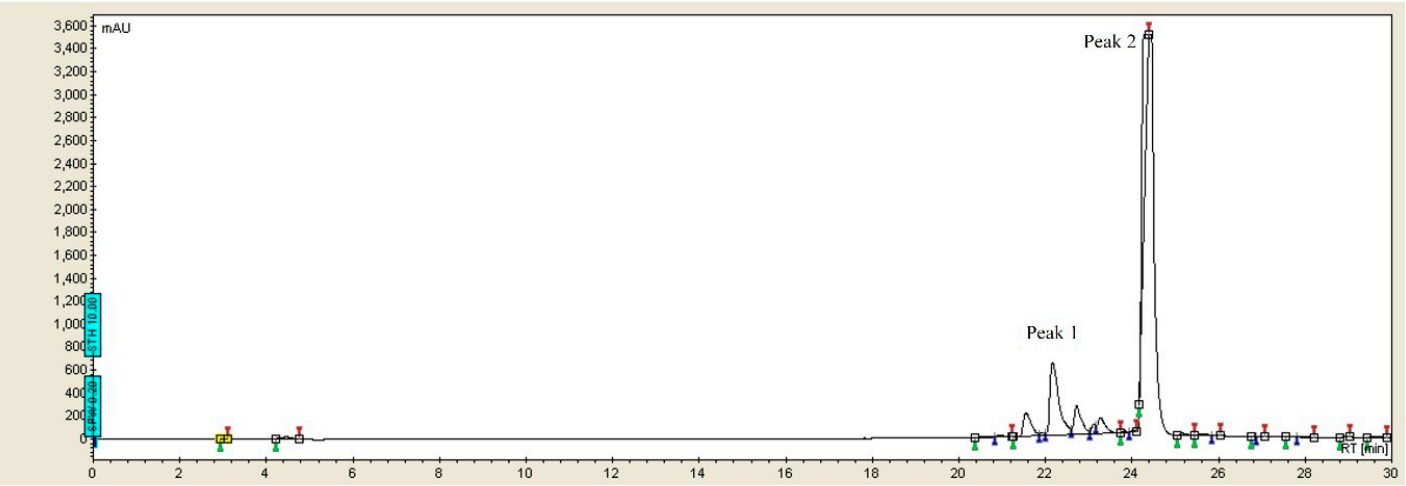

Figure S2. HPLC chromatogram of BP-C1II-C2IV-SPEIV; peak 1 = unidentified compound, peak 2 = mixture of squamin C + squamin D (co-eluting compounds), monitored at 230 nm.

Table S1. 1H and 13C NMR spectroscopic data for cherimolacyclopeptide D (CD_3OD, 298 K).

Amino acid	Position	δ_C	δ_H, mult. (*J* in Hz)
Pro[1]	CO	174.7	
	αCH	62.7	4.26, m
	βCH_2	30.6	1.95, m<br>2.26, td (6.4, 3.0)
	γCH_2	26.1	1.98, m<br>2.15, m
	δCH_2	49.9	3.73, td (9.6, 6.7)<br>4.23, m
Gly[2]	CO	171.1	
	NH		8.88, dd (8.7, 4.1)
	αCH_2	43.8	3.48, dd (17.2, 4.1)<br>4.29, m
Leu[3]	CO	173.9	
	NH		8.18, d (10.4)
	αCH	54.8	4.75, ddd (10.5, 8.4, 6.7)
	βCH_2	44.9	1.39, m<br>1.53, m
	γCH	25.9	1.55, m
	δCH_3	22.7	0.95, d (6.4)
	$\delta' CH_3$	22.8	0.91, d (6.3)
Asn[4]	CO	172.8	
	NH		8.39, d (5.7)
	αCH	51.8	4.57, dq (6.1, 2.6)
	βCH_2	35.6	3.27, m
	NH		7.56, s
	NH		8.12, s
	CO	175.3	
Ala[5]	CO	175.3	
	NH		8.34, d (4.1)
	αCH	53.4	4.13, qd (7.3, 3.8)
	βCH_3	17.4	1.44, d (7.4)
Val[6]	CO	173.6	
	NH		8.14, d (10.3)
	αCH	61.5	4.27, m
	βCH	30.6	2.17, m
	γCH_3	19.3	0.91, d (6.7)
	$\gamma' CH_3$	19.7	0.96, d (6.7)
Thr[7]	CO	170.7	
	NH		8.25, d (9.0)
	αCH	58.5	4.66, t (9.2)
	βCH	68.5	3.83, dq (9.6, 6.3)
	γCH_3	20.4	1.20, d (6.4)

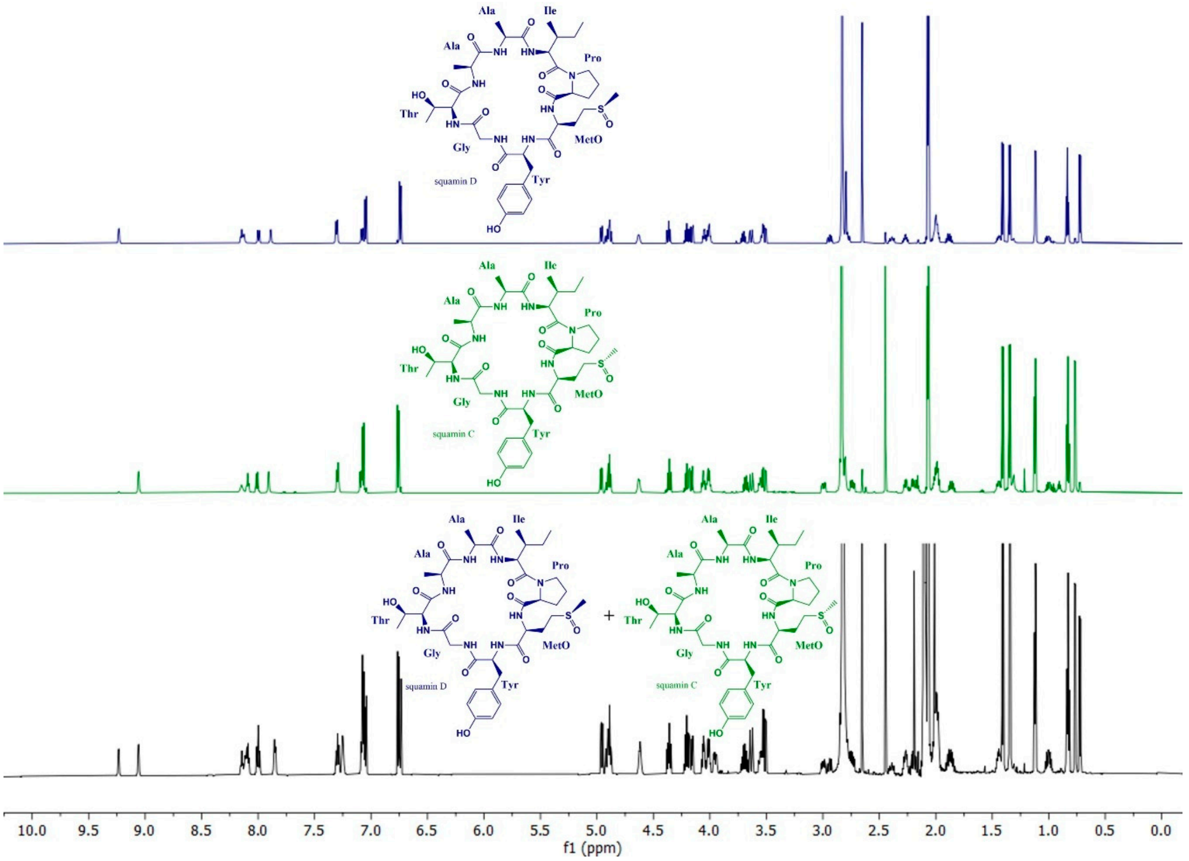

Figure S3. Stacked ^{1}H-NMR spectra of the mixture of squamins C + D (black), compared with pure squamin D (blue) and pure squamin C (green), in acetone-d_6 at 700 MHz (pure compound spectra from Murrieta-Dionicio et al., 2024).

Supplementary material of Docking analysis

Table S2. Docking cavity screening parameters for all evaluated cavities.

Receptor	Ligand	Pocket	Vina score	Volume (Å³)	Center (x,y,z)	Box (x,y,z)	Selected	Rationale
5-HT$_{1A}$	CPD	C1	-8.7	3619	93, 67, 66	29, 35, 35	No	Best score but less suitable for comparative analysis
5-HT$_{1A}$	CPD	C4	-7.2	1916	99, 120, 113	23, 23, 23	Yes	Selected biologically plausible/common region
5-HT$_{1A}$	CPD	C3	-7.0	2720	93, 98, 66	32, 29, 30	No	
5-HT$_{1A}$	CPD	C2	-6.6	3192	78, 49, 65	23, 23, 31	No	
5-HT$_{1A}$	CPD	C5	-6.2	1778	94, 101, 86	23, 23, 23	No	
5-HT$_{1A}$	SQC	C4	-10.1	1916	99, 120, 113	24, 24, 24	Yes	Best score and selected region
5-HT$_{1A}$	SQC	C2	-7.7	3192	78, 49, 65	24, 24, 31	No	
5-HT$_{1A}$	SQC	C3	-7.2	2720	93, 98, 66	32, 24, 30	No	
5-HT$_{1A}$	SQC	C5	-6.9	1778	94, 101, 86	24, 24, 24	No	
5-HT$_{1A}$	SQC	C1	-3.0	3619	93, 67, 66	24, 35, 35	No	

5-HT$_{1A}$	SQD	C3	-9.3	2720	93, 98, 66	32, 24, 30	No	Best score but not used for cross-ligand comparison
5-HT$_{1A}$	SQD	C4	-8.7	1916	99, 120, 113	24, 24, 24	Yes	Selected biologically plausible/common region
5-HT$_{1A}$	SQD	C5	-8.3	1778	94, 101, 86	24, 24, 24	No	
5-HT$_{1A}$	SQD	C2	-8.0	3192	78, 49, 65	24, 24, 31	No	
5-HT$_{1A}$	SQD	C1	-7.0	3619	93, 67, 66	24, 35, 35	No	
GABA$_A$	CPD	C3	-7.5	946	43, 10, 39	29, 23, 35	No	Best score; reported in supplement
GABA$_A$	CPD	C5	-7.3	678	63, 1, 60	23, 23, 23	Yes	Selected for common C5 comparison with SQC/SQD
GABA$_A$	CPD	C4	-6.8	741	35, -5, 28	23, 23, 30	No	
GABA$_A$	CPD	C2	-6.6	1011	54, 13, 34	23, 23, 35	No	
GABA$_A$	CPD	C1	-5.1	1559	15, -14, 7	23, 23, 23	No	
GABA$_A$	SQC	C5	-9.9	678	63, 1, 60	24, 24, 24	Yes	Best score and selected region
GABA$_A$	SQC	C3	-7.7	946	43, 10, 39	24, 24, 35	No	
GABA$_A$	SQC	C4	-7.3	741	35, -5, 28	24, 24, 30	No	
GABA$_A$	SQC	C2	-7.0	1011	54, 13, 34	24, 24, 35	No	
GABA$_A$	SQC	C1	-5.8	1559	15, -14, 7	24, 24, 24	No	
GABA$_A$	SQD	C5	-9.1	678	63, 1, 60	24, 24, 24	Yes	Best score and selected region
GABA$_A$	SQD	C1	-7.3	1559	15, -14, 7	24, 24, 24	No	
GABA$_A$	SQD	C2	-7.3	1011	54, 13, 34	24, 24, 35	No	
GABA$_A$	SQD	C3	-7.3	946	43, 10, 39	24, 24, 35	No	
GABA$_A$	SQD	C4	-6.8	741	35, -5, 28	24, 24, 30	No	

Table S3. Principal contact residues used for interpretation and figure labeling.

Receptor	Ligand	Cavity	Principal contact residues
5-HT$_{1A}$	CPD	C4	Tyr96, Gln97, Asn100, Thr188, Ile189, Phe361, Val364, Asn386
5-HT$_{1A}$	SQC	C4	Tyr96, Gln97, Asn100, Asp116, Thr188, Ile189, Phe361, Phe362, Val364, Gly382, Ala383, Asn386, Trp387, Tyr390
5-HT$_{1A}$	SQD	C4	Tyr96, Gln97, Asn100, Phe112, Asp116, Thr188, Ile189, Phe361, Val364, Gly382, Asn386, Tyr390
GABA$_A$	CPD	C5	Val256, Val259, Thr260, Leu263, Thr264, Thr267
GABA$_A$	SQC	C5	Val256, Val259, Thr260, Leu263, Thr264, Thr267; Met265/Leu268 in the broader C5 environment
GABA$_A$	SQD	C5	Val256, Val259, Thr260, Leu263, Thr264, Thr267; Met265/Leu268 in the broader C5 environment

Table S4. Docking-derived Ki and pKi values for selected poses.

Receptor	Ligand	Cavity	ΔG (kcal/mol)	Ki (M)	Ki	pKi

5-HT$_{1A}$ (7E2Y)	CPD	C4	-7.2	5.28e-06	5.28 μM	5.28
5-HT$_{1A}$ (7E2Y)	SQC	C4	-10.1	3.95e-08	39.5 nM	7.40
5-HT$_{1A}$ (7E2Y)	SQD	C4	-8.7	4.20e-07	420 nM	6.38
GABA$_A$ chimera (5OSA)	CPD	C5*	-7.3	4.46e-06	4.46 μM	5.35
GABA$_A$ chimera (5OSA)	SQC	C5	-9.9	5.54e-08	55.4 nM	7.26
GABA$_A$ chimera (5OSA)	SQD	C5	-9.1	2.14e-07	214 nM	6.67
